# Factors Associated With Length of Stay and Readmission Rates for Older Hospital in the Home Patients: A Systematic Review

**DOI:** 10.1177/08982643251329425

**Published:** 2025-03-27

**Authors:** Kerry de Vent, Joanne E. Porter, Jo-ann Larkins

**Affiliations:** 1 Collaborative Evaluation and Research Centre (CERC), 72534 Federation University Australia, Churchill, VIC, Australia

**Keywords:** HITH, hospital in the home, older adults, geriatric, home-based care

## Abstract

**Objective:**

The aim of this study is to conduct a systematic review of published literature to examine factors associated with Length of Stay (LOS) and Readmission Rates for older Hospital in the Home (HITH) patients.

**Method:**

In accordance with PRISMA guidelines, seven databases were searched for peer-reviewed articles relating to HITH, older patients, LOS and readmissions.

**Results:**

Twenty-nine studies met the inclusion criteria. Risk factors associated with increased readmissions and LOS were age, prior hospitalisations, illness severity, geriatric-related complications, and cognitive impairment. Most studies found that patients participating in HITH had a shorter initial acute hospitalisation LOS than patients transferred to a subacute hospital or rehabilitation ward. However, LOS and readmissions, comparing HITH to traditional in-hospital care, were inconsistent.

**Conclusions:**

Overall, LOS and readmission rates (comparing home-based care to hospital care) were inconsistent but appear related to patient demographics and disease profile and require further study.

## Introduction

Ageing populations in developed societies represents a challenge for health service providers (Closa et al., 2017) with elderly patients more likely to present with complex clinical presentations and chronic conditions requiring multidisciplinary interventions (De Stampa et al., 2023). This is due to increased frailty, disability and comorbidities associated with advanced age (Mas et al., 2017). In the process of treating elderly patients, care delivered in an acute hospital setting can have adverse effects, particularly Iatrogenic illnesses, functional decline, delirium, and other adverse outcomes. Hospital care can also be expensive and disruptive not only to patients, but carers and families alike (Leff et al., 1999; Mader et al., 2008). Long episodes of acute hospital stay may also impact a patient’s ability to adjust to life at home following discharge (Mas et al., 2017).

To address these issues, a promising alternative to acute-based hospital care is Hospital in the Home (HITH), also referred to as Hospital at Home (HAH) or home hospital (HH) which is a model of care which enables patients to receive acute care at home. HITH can entirely replace an in-hospital stay or enable patients to receive post-hospitalisation care at home (De Stampa et al., 2023). HITH enables the provision of hospital-equivalent care at home (Cai et al., 2018) and has shown to be effective, with a high degree of patient and caregiver satisfaction (De Stampa et al., 2023). There are different modes of HITH, namely admission avoidance and early assisted discharge. Admission avoidance is where patients are treated in their own home instead of an acute hospital setting, offering a true alternative to standard hospitalisation (Crisci, 2023) whereas early assisted discharge is when a patient is initially treated in hospital and then continues their treatment at home.

HITH may have implications for both Length of Stay (LOS) in hospital and readmissions to hospital after discharge. These can be used as indicators of hospital performance and are closely related. This systematic review aims to describe factors relating to LOS and readmissions for older patients receiving home-based treatment such as HITH. This systematic review is necessary as HITH-programs are increasingly being used for older patients worldwide, however, the impact of HITH on hospital performance indicators such as LOS and readmissions are varied. This review consolidates findings from studies to allow a greater understanding of these outcomes and allows comparisons between HITH and traditional hospitalisations in older patients. An understanding of the impact of HITH on LOS and readmissions can help healthcare policymakers and administrators make informed decisions about implementing HITH programs for older patients.

## Method

### Protocol and Registration

A systematic review was undertaken according to the PRIMSA (Preferred Reporting Items for Systematic Reviews and meta-Analysis) statement (Page et al., 2021). The systematic review was registered in the PROSPERO register of systematic reviews (registration number: CRD42024544908). There were no other protocols relating to LOS and Readmission Rates for older HITH patients at the time of registration. There was one amendment to the protocol that was registered initially, namely the title, which has been changed from ‘patients aged 65 and over’ to ‘older Hospital in the Home patients’, to enable use of articles that reference ‘older patients’ without necessarily specifying ‘over 65’.

### Search Strategy

PRISMA guidelines were used to conduct a systematic search using the databases: SCOPUS, MEDLINE, CINAHL complete, APA PsycInfo, APA PsycArticles, and Web of Science. Additional articles were identified by screening the reference lists of selected articles. Key search terms relating to ‘home care’ or HITH, older patients, and Length of Stay (LOS) and readmissions were used and synonyms and closely related words were included. Refer to supplementary table for full search strategy. There were no date limitations imposed on database search.

## Inclusion and/or Exclusion Criteria

All database records were loaded into Covidence, (Covidence, 2023) and duplicates were removed based on title and author. The title and abstract screening were performed by two reviewers (author 1 and author 2). Both authors assessed each article independently, reaching consensus in the vast majority of cases (>80%). Disagreements between the reviewers were resolved by discussion between author 1 and author 2, in all cases leading to consensus. After articles were selected, they were imported into EndNote 21.0.1 (The EndNote Team, 2024) where full text was retrieved and full text screening was performed. Study participants were older patients with any disease/reason for hospitalisation. In general, a definition of ‘older patients’ includes patients aged 65 and over, but for completeness of this study, this definition was relaxed to include articles that report ‘older patients’ and those with a mean age of over 65, which may include some younger patients. Studies were selected if they reported on LOS and readmission for older patients, focusing on HITH. Only peer-reviewed articles were considered. Refer to Table 1 for inclusion/exclusion criteria.

**Table 1.** Inclusion and Exclusion Criteria.

Inclusion criteria	Exclusion criteria
Studies that report on length of stay and readmission	Studies with no mention of length of stay and readmission
Healthcare organisations such as hospital, clinics or hospital departments	Unpublished research, letters to the editor, conference abstracts, non-peer reviewed articles and dissertations
Older patients	Paediatric patients, young patients
Focus on HITH (hospital in the home)	Commentary, conference proceedings
Peer reviewed primary literature	Grey literature, unpublished works
All study designs with clear methodology	

## Quality Appraisal

Risk of bias was assessed using CASP (Critical Appraisal Skills Programme, 2024) using two checklists, one for RCT and the other for cohort and other study designs (see Table 2 and Table 3). The scores ranged from 75% to 100%. A score of 75% was chosen as the cut-off for quality assessment and no articles were excluded based on risk of bias assessment. Author 1 undertook risk of bias assessment.

**Table 2.** CASP Checklist for Randomised Control Trials Randomised Control Trials CASP Checklist.

	1	2	3	4a*	4b*	4c*	5	6	7	8	9	10	11	%
(Aimonino Ricauda et al., 2008)	✓	✓	✓			✓	✓	✓	✓	✓	✓	✓	✓	85
(Caplan et al., 2006)	✓	✓	✓		✓		✓	✓	✓	✓	✓	✓	✓	85
(Davies et al., 2000)	✓	✓	✓		✓		✓	✓	✓	✓	✓	✓	✓	85
(Hernandez et al., 2003)	✓	✓	✓		✓		✓	✓	✓	✓	✓	✓	✓	85
(Jones et al., 1999)	✓	✓	✓				✓	✓	✓	✓	✓	✓	✓	77
(Mendoza et al., 2009)	✓	✓	✓				✓	✓	✓	✓	✓	✓	✓	77
(Messecar, 1999)	✓	✓	✓				✓	✓	✓	✓	✓	✓	✓	77
(Shepperd et al., 2022)	✓	✓	✓				✓	✓	✓	✓	✓	✓	✓	77
(Tibaldi et al., 2009)	✓	✓	✓		✓		✓	✓	✓	✓	✓	✓	✓	85

*These questions related to blinding. Due the nature of the studies, it was impossible to administer a double blind trial, however in some cases single blinding was used.

**Table 3.** CASP Checklist for Other Experimental Designs Other Study Designs CASP Checklist.

	1	2	3	4	5a	5b	6a	6b	9	10	11	12	%
(Anderson et al., 1999)	✓	✓	✓	✓			✓	✓	✓	✓	✓	✓	83
(Arias-de La Torre et al., 2020)	✓	✓	✓	✓			✓	✓	✓	✓	✓	✓	83
(Augustine et al., 2023)	✓	✓	✓	✓	✓	✓	✓	✓	✓		✓	✓	92
(Bori et al., 2010)	✓	✓	✓	✓			✓	✓	✓		✓	✓	75
(Chia et al., 2020)	✓	✓	✓	✓			✓	✓	✓	✓	✓	✓	83
(Closa et al., 2017)	✓	✓	✓	✓			✓	✓	✓	✓	✓	✓	83
(De Stampa et al., 2023)	✓	✓	✓	✓			✓	✓	✓	✓	✓	✓	83
(Eeles et al., 2016)	✓	✓	✓	✓			✓	✓	✓	✓	✓	✓	83
(Leff et al., 1999)	✓	✓	✓	✓			✓	✓	✓		✓	✓	75
(Leff et al., 2005)	✓	✓	✓	✓			✓	✓	✓		✓	✓	75
(Lim et al., 2021)	✓	✓	✓	✓			✓	✓	✓	✓	✓	✓	83
(Loveland et al., 2022)	✓	✓	✓	✓			✓	✓	✓	✓	✓	✓	83
(Mader et al., 2008)	✓	✓	✓	✓			✓	✓	✓		✓	✓	75
(Mas et al., 2017)	✓	✓	✓	✓	✓	✓	✓	✓	✓	✓	✓	✓	100
(Montalto et al., 2020)	✓	✓	✓	✓			✓	✓	✓	✓	✓	✓	83
(Morgan et al., 2019)	✓	✓	✓	✓			✓	✓	✓	✓	✓	✓	83
(O’Cathain, 1994)	✓	✓	✓	✓	✓	✓	✓	✓	✓	✓	✓	✓	100
(Tierney et al., 2021)	✓	✓	✓	✓	✓		✓	✓	✓	✓	✓	✓	92
(Vilà et al., 2015)	✓	✓	✓	✓			✓	✓	✓	✓	✓	✓	83
(Yu & Sunderland, 2020)	✓	✓	✓	✓	✓	✓	✓	✓	✓	✓	✓	✓	100

### Synthesis of Results

The studies were deemed too heterogeneous to statistically combine because there were differences in study design, particularly the way they measured LOS and readmissions (such as differences in the follow up period). Therefore a narrative synthesis (systematic review without meta-analysis) was used.

## Results

**Figure 1.**
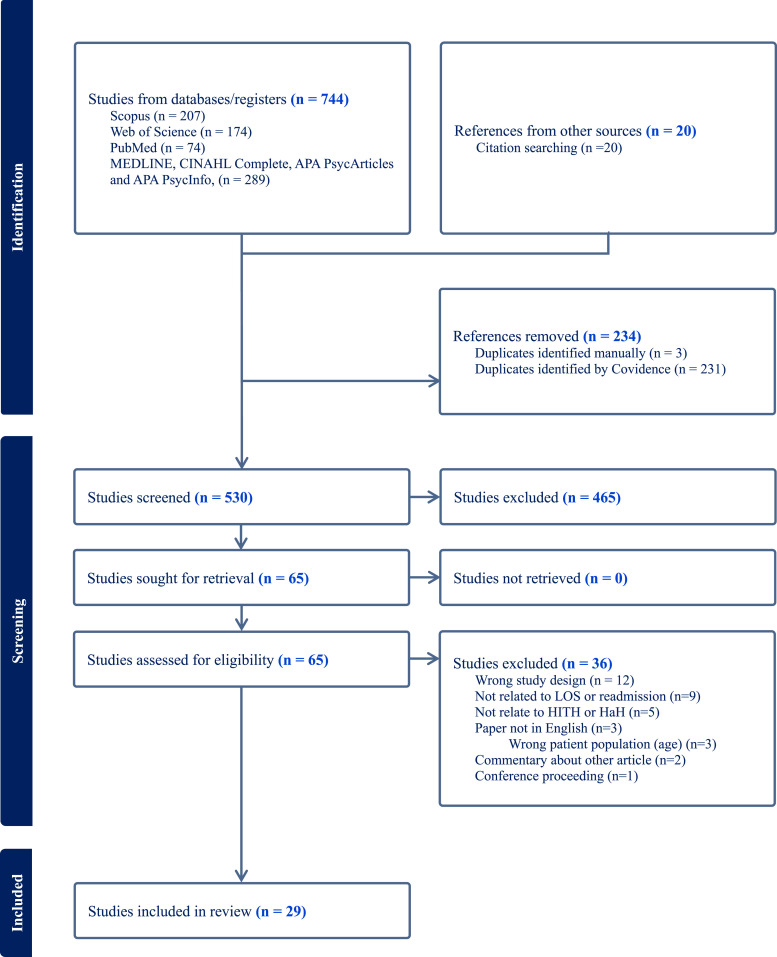
PRISMA flow diagram.

The initial search found 744 articles from databases and 20 articles found by screening references of articles. A total of 234 duplicates were identified by Covidence and 3 duplicates were manually identified. Based on title and abstract screening, 465 studies were excluded, leaving 65 articles to screen using analysis of full text. Based on full text screening a further 36 articles were excluded, leaving 29 articles to include in this review. Refer to Figure 1 for PRIMSA diagram.

### Study Characteristics

The studies included in this review were from a variety of countries. Seven were from Australia, seven were from Spain, six were from England, five were from the U.S., two were from Italy, one was from France and one was from Ireland. Out of the 29 studies, nine were randomised control trials (RCTs), with the other studies being retrospective reviews, cross-sectional studies, and other study designs. See Table 4 for study characteristics.

**Table 4.** Study Characteristics.

Author, year	Location	Study design	Sample size and age	Purpose of the study
(Aimonino Ricauda et al., 2008)	Italy	Prospective randomised, controlled trial with 6 month follow up	*N* = 104 Age 75+	Hospital readmission rates and mortality for elderly patients with exacerbation of COPD.
(Anderson et al., 1999)	U.S.	Retrospective medical record review	*N* = 68 Age 65+	To describe patients who have returns to an inpatient setting during the first 100 days of home care delivery
(Arias-de La Torre et al., 2020)	Spain	Exploratory cross-sectional study	*N* = 9601 Mean age 69.9 (admission avoidance) and 69.6 (early discharge)	Describe characteristics of HITH, assess results and factors
(Augustine et al., 2023)	U.S.	Comparative effectiveness analysis	*N* = 5708 majority 65+ (only 3.5% under the age of 65)	Effectiveness of RaH (rehabilitation at home)
(Bori et al., 2010)	Spain	Descriptive study	*N* = 47 Mean age = 65	Evaluate complications and LOS associated with primary unilateral him arthroplasty undergoing new protocol of home-based specialised care
(Caplan et al., 2006)	Australia	Randomised control trial	*N* = 104 Mean age home = 83.86 Mean age hospital = 84.00	Rehabilitation of elderly care at home compared to usual treatment, and effect on delirium
(Chia et al., 2020)	Australia	Retrospective chart review	*N* = 96 Age 65+	Evaluate mortality, readmission and efficiency (LOS) HITH delirium pathway compared with hospital-based care
(Closa et al., 2017)	Spain	Quasi-experimental longitudinal study	*N* = 367 Age 65+	Compare outcomes and costs in home-based care versus hospital-based care, for patients with orthogeriatric conditions
(Davies et al., 2000)	England	Prospective randomised controlled trial	*N* = 150 Mean age = 70	Compare HITH and hospital care for exacerbation of COPD.
(De Stampa et al., 2023)	France	3-year retrospective study	*N* = 75108 Age 75+	Readmission predictors for HITH patients
(Eeles et al., 2016)	Australia	Before-after prospective study	*N* = 24 Age 65+	Evaluation of HITH for patients with delirium
(Hernandez et al., 2003)	Spain	Randomised control trial	*N* = 222 Mean age = 71	Evaluate home hospitalisation of patients with COPD
(Jones et al., 1999)	England	Cost minimisation analysis within a pragmatic randomised controlled trial	*N* = 199 Median age = 84	Compare costs between home hospital scheme with acute hospital admission
(Leff et al., 1999)	U.S.	Prospective case series	*N* = 139 Age 65+	Evaluate home hospital (safety and feasibility) for treating acutely ill older patients
(Leff et al., 2005)	U.S.	Prospective quasi-experiment	*N* = 455 Age 65+	Assess feasibility and efficacy of hospital at home
(Lim et al., 2021)	Australia	Retrospective cohort study	*N* = 605 Mean age = 56	Study the incidence of readmissions and risk factors for readmission in a HITH programme
(Loveland et al., 2022)	Australia	Observational longitudinal cohort study	*N* = 92 Age 65+	Describe home based rehabilitation model for older patients
(Mader et al., 2008)	U.S.	Descriptive study	*N* = 225 Mean age = 68.7	Describe process used to transition the HITH model at the Portland VA medical centre
(Mas et al., 2017)	Spain	Quasi-experimental longitudinal study	*N* = 849 Mean age = 83.2	Compare home-based care model with usual bed-based care, and analyse the clinical impact
(Mendoza et al., 2009)	Spain	Prospective, randomized study	*N* = 71 Age 65+	Compare HITH to inpatient care for elderly patients with decompensated heart failure
(Messecar, 1999)	England	Randomised controlled trial	*N* = 241 Median age = 79	Effectiveness of HITH compared to inpatient hospital care for medically stable older patients
(Montalto et al., 2020)	Australia	Descriptive, retrospective study	*N* = 2,185,421 Age = large proportion aged 50–79 years	Describe uptake of HIH, and characteristics of patients and their HIH admissions
(Morgan et al., 2019)	England	Retrospective study	*N* = 108 Age 65+	Comparison of 1 year patient outcomes following admission to HITH or hospital for COPD exacerbation
(O’Cathain, 1994)	England	Retrospective study	*N* = 110 Mean age HAH = 76.4, Hospital = 77.6	Evaluation of HITH for early discharge of patients with fractured neck of femur
(Shepperd et al., 2022)	England	Randomised control trial	*N* = 1055 Age 65+	Clinical and cost-effectiveness of admission avoidance at home: the experiences of older patients and caregivers ,and how the services differed
(Tibaldi et al., 2009)	Italy	Randomised controlled trial	*N* = 101 Age 75+	Feasibility and effectiveness of physician-led HITH service for elderly patients with CHF.
(Tierney et al., 2021)	Ireland	Retrospective chart review. Quantitative design, using service evaluation methodology	*N* = 505 Mean age HAH = 83.5, Hospital = 84.2	Impact of acute care at home service in comparison with conventional hospital admission for elderly patients
(Vilà et al., 2015)	Spain	Retrospective study	*N* = 261 Mean Age = 84	Investigate cost-effectiveness of home care program for patient with multimorbidity
(Yu & Sunderland, 2020)	Australia	Retrospective cohort study	*N* = 128 Age 65+	Readmission, mortality and complications in older patients with CCF, compare HITH to those treated in hospital

### Length of Stay: Comparing Home-Based Hospitalisation to Traditional In-Hospital Care

Six studies (Augustine et al., 2023; Bori et al., 2010; Caplan et al., 2006; Chia et al., 2020; Closa et al., 2017; Jones et al., 1999) examined patients who experienced an initial stay in hospital and were subsequently transferred either to a sub-acute/rehabilitation ward or to home-based hospital (HITH). These studies included a variety of conditions treated and various hospital sizes (see Table 5 for details). All studies except one (Caplan et al., 2006) found that patients transferred to home-based hospital were associated with a reduction in the initial time spent in acute based hospital care. Caplan et al. (2006), however, found that patients receiving home-hospitalisation spent an average extra 2 days in an acute ward, however, overall LOS from hospital admission to end of rehabilitation was not significantly different between the hospital rehabilitation ward and the home hospital.

**Table 5.** Synthesis of Results: Length of Stay Comparison Between Home Based Hospitalisation and Traditional in Hospital Treatment.

Study	HITH Mean	HITH SD	HITH *N*	Hospital Mean	Hospital SD	Hospital *N*	Condition
LOS in acute care before being transferred to post-acute hospital ward or home-based care							
(Augustine et al., 2023)	8.1	7.6	173	8.7	11.2	173	All conditions
(Bori et al., 2010)	3.7	1	47	4.59	0.68	47	Total hip arthroplasty
(Caplan et al., 2006)	18.73	11.39	70	17.03	8.68	34	Delirium
(Chia et al., 2020)	7.7	11.1	46	10.8	12.1	50	Delirium
(Closa et al., 2017)	10.1	7	91	15.3	12	276	Orthogeriatric
(Jones et al., 1999)	5.1	13.53	102	18.5	18.51	97	All conditions
LOS in home-based care versus in-patient hospital care							
(Aimonino Ricauda et al., 2008)	15.5	9.5	41	11	7.9	39	COPD
(Eeles et al., 2016)	10.8	13.8	16	12.5	12.5	8	Delirium
(Hernandez et al., 2003)	1.71	2.33	121	4.15	4.1	101	COPD
(Leff et al., 1999)	2.9	1.5	17	3.4	2.5	122	Community-acquired pneumonia, chronic heart failure, chronic obstructive airways disease, or cellulitis
(Leff et al., 2005)	3.2	2.5	169	4.9	9.9	286	Community-acquired pneumonia, exacerbation of chronic heart failure, exacerbation of chronic obstructive pulmonary disease, or cellulitis
(Mas et al., 2018)	9.6	3.9	57	8.3	2.9	114	Complex chronic conditions

The articles described above considered LOS in acute care followed by admission to either sub-acute/rehabilitation ward or to home-based care. It has been argued that this type of HITH model (early-discharge) may be of questionable value, with early-discharge model allowing patients to complete some of their treatment already started in hospital. In these cases, patients are often well past the acute phase and the early-discharge HITH may be somewhat a glorified ‘discharge-to-home’ programme which may not be cost-effective, unlike ‘true-HITH’ (Crisci, 2023).

The other type of HITH model is ‘admission avoidance’ where home-based care completely replaces an in-hospital stay. Six articles directly compared LOS to home-based care, where patients were directly admitted to one or the other (from an emergency department, or from a GPs office) without an initial hospital stay (Aimonino Ricauda et al., 2008; Eeles et al., 2016; Hernandez et al., 2003; Leff et al., 1999, 2005; Mas et al., 2018). In terms of LOS, when comparing traditional hospital care to home-based hospital, the results were inconsistent. Four studies found that in-hospital care was associated with longer LOS, with two studies contradicting this finding (See Table 5 for details).

### Readmission: Comparing Home-Based Hospitalisation to Traditional In-Hospital Care

There were 13 studies in this review that reported readmission rates (see Table 6). There were inconsistent results, with seven studies reporting that in-hospital care was associated with increased readmission rates (Aimonino Ricauda et al., 2008; Caplan et al., 2006; Hernandez et al., 2003; Mendoza et al., 2009; Morgan et al., 2019; Tibaldi et al., 2009; Yu & Sunderland, 2020) and other studies reporting the opposite (Chia et al., 2020; Davies et al., 2000; O'Cathain, 1994; Tierney et al., 2021). Readmission rates were examined for a variety of conditions, and had different follow-up periods ranging from 30 days to 1 year.

**Table 6.** Synthesis of Results: Readmission Rates (%) Comparison Between Home Based Hospitalisation and Traditional in Hospital Treatment.

Study	Home based hospital readmission	Traditional in-hospital readmission	*p* value	Condition	Follow up period
(Aimonino Ricauda et al., 2008)	42%	87%	0.001	COPD	6 month
(Caplan et al., 2006)	21%	24%	1.00	Delirium	28 day
(Chia et al., 2020)	30%	18%	0.23	Delirium	1 month
(Davies et al., 2000)	37%	34%	Not stated	COPD	3 month
(De Stampa et al., 2023)	28%		*N*/A	All conditions	30 day
(Eeles et al., 2016)	25%	25%	1.00	Delirium	1 year
(Hernandez et al., 2003)	20%	28%	Not stated	COPD	8 week
(Mendoza et al., 2009)	41%	50%	0.42	Heart failure	1 year
(Morgan et al., 2019)	8%	20%	Not stated	COPD	6 month
(O’Cathain, 1994)	16%	9%	Not sig	Fracture neck of femur	3 month
(Tibaldi et al., 2009)	17%	34%	0.19	Chronic heart failure	6 month
(Tierney et al., 2021)	9%	3%	0.015	All conditions	30 day
(Yu & Sunderland, 2020)	20%	23%	0.7	Acute decompensated congestive cardiac failure	30 day
(Yu & Sunderland, 2020)	33%	41%	0.7	Acute decompensated congestive cardiac failure	60 day

### Safety and Cost-Effectiveness of HITH

HITH for home-based care and rehabilitation has been found to be as effective and safe compared to in-hospital care, and may have cost-benefits (Arias-de La Torre et al., 2020; Closa et al., 2017; Loveland et al., 2022; Yu & Sunderland, 2020). Tierney et al. (2021) reported that care given at home may be more cost-effective than inpatient care. Home treatment has been shown as safe, feasible and effective with cost reductions, perhaps due to decreased LOS and lower levels of healthcare utilization (Johnson, 2021). In terms of safety and efficacy, mortality rate can be used as a clinical outcome. Tierney et al. (2021) found that although almost all studies reviewed showed no difference in mortality rates between home-based and hospital-based care, their particular study reported an increase in mortality for home-based patients. This may be attributed to the fact that people using the HITH service are more likely to be frail, dependent, and of very old age (De Stampa et al., 2023). Patients admitted to home-based rehabilitation are predominantly older and present with more comorbidities and cognitive impairment, however despite this, most patients completed their rehabilitation at home with improved physical performance, better mobility and improved functional independence and frailty (Loveland et al., 2022). Cryer et al. (2012) found that patients treated at home had comparable or even better outcomes than in-hospital patients and presented with higher satisfaction levels. In a study of chronic HF, the HITH model showed no significant differences in functional or clinical outcomes but was associated with a reduction in direct costs (Mendoza et al., 2009). Although Tierney et al. (2021) reported higher rates of mortality (at 30 days, 3 months and 6 months), their study found that fewer patients died while under the care of home-based services than traditional hospital care. They reported that home-based care appears equally safe compared with hospital-based care when treating older patients.

In terms of safety, HITH may reduce adverse events such as nosocomial infections and psychological distress, as patients may feel more comfortable with treatment at home, with an improvement in overall quality of life (Lim et al., 2021). Patients favour receiving hospital care at home where they can move around their home and sleep in their own beds during their recovery. Home-based hospital care was associated with no differences in rates of adverse consequences, in fact some studies found that home-hospital was associated with fewer clinical complications. Patients treated at home report a beneficial increase in physical activity, and less time in bed which can help prevent events such as blood clots and pressure sores (Johnson, 2021). In terms of disease escalation (e.g. necessitating a readmission), Hernandez et al. (2023) found that home based hospital had a similar escalation rate than traditional hospital care, and no mortality during home care.

### Discussion and Implications

Home-based hospital care may be a worthwhile alternative to traditional in-hospital care, particularly for older patients (Aimonino Ricauda et al., 2008) and may influence LOS and rates of hospital readmission, however, the results were inconsistent. This highlights the need to explore factors which underlie LOS and readmissions for HITH patients.

In the studies examining home-based care after an initial acute hospital stay, most found that the initial hospital stay was shorter for patients transferred to home-hospital care compared to patients transferred to sub-acute in-hospital care or rehabilitation. A reason for this may be fewer delays in transferring the patient. For patients transferred to a hospital rehabilitation ward, there may be delays in securing a bed. Home hospital patients have the benefit of not having to wait for a bed to become available in a post-acute or rehabilitation ward. Caplan et al. (2006) was the only study that found initial (acute) hospital stay was longer for patients receiving home-hospital care. A reason for this inconsistency may be that patients are required demonstrate higher levels of functional independence before transferred to home care, which may not be necessary when being transferred to a hospital rehabilitation facility.

In comparing LOS and readmission rates between HITH and in-hospital care (admission avoidance model), the results were inconsistent however some themes emerged which may help explain these inconsistencies. It appears that the type of patient being treated and disease type and severity may influence on both LOS and readmission rate.

Age of the patient appears to be an important predictor for LOS and readmissions, as does the disease and treatment regime. Anderson et al. (1999) conducted a pilot study focussing on older patients readmitted within the first 31 days of receiving home hospital care. Patients who were readmitted were predominantly older. This was also reflected in a study by Lim et al. (2021) who found that patients readmitted were on average 6.9 years older than patients who were not readmitted. Old age is often associated with increased cognitive impairment, and delirium, as well as the prevalence of geriatric-related conditions which may explain the association of increased age with increased readmission (Mas et al., 2017). In terms of type of care, those receiving intravenous treatment, postsurgery treatment were less likely to be readmitted and patients receiving supportive cancer treatment were more likely to be readmitted (De Stampa et al., 2023). Other factors associated with readmission included patients referred from the ED, recent ICU admission, high scores on the CCI (Charlson Comorbidity Index), advanced chronic kidney disease, patients treated with negative pressure wound therapy, and use of hypertensives (Lim et al., 2021).

The overall reasons for unplanned hospital readmission were worsening of the primary diagnosis (37.9%) worsening of the secondary diagnosis (18.3%) or development of a new problem (Anderson et al., 1999). Lim et al. (2021) identified five main categories associated with hospital readmissions. These include treatment failure, which includes cases where there was a lack of improvement or deterioration of the initial condition requiring HITH admission. Secondly, some readmissions may be attributed to development of a complication related to the initial conditions, or side effects related to treatment. Thirdly, there may be development of a new condition unrelated to the initial condition. There may also be functional decline or psychosocial concerns, and finally readmission may be associated with planned surgical procedures.

Some studies found reduced LOS and fewer readmissions for HITH patients. In these cases, patient selection may influence these findings. In many cases patients need to be medically stable to be transferred to home-based hospital care and may experience less severe health conditions or co-morbidities. In some cases, patients were ineligible for home-based care if they were acutely ill as decided by the treating clinician (Chia et al., 2020). The fact that less severely ill patients are being admitted to HITH may contribute to the findings of lower LOS and fewer readmissions. HITH may lead to better patient outcomes, such as reduced mortality and lower rates of readmission, however, preferred candidates for HITH may involve patients who are expected to experience good outcomes anyway (Montalto et al., 2020). Selecting patients based on robust eligibility criteria is important to ensure that patients treated as HITH may benefit from this model of care (Voudris & Silver, 2018). Additionally, home-based care may be associated with decreased LOS due to lower rates of infection (particularly nosocomial infections) and other complications that are associated with longer hospital stays. These patients enjoy greater freedom, such as not having to adhere to a hospital routine, which may have inherent health and wellbeing benefits. Being in a familiar and comfortable environment may lead to a faster recovery (Cai et al., 2018).

Some studies contradicted these results and found that HITH patients were associated with poorer outcomes such as increased LOS and higher readmission rates. Again, this may be due to the type of patient being admitted to HITH. In some cases HITH patients tended be older (Achanta et al., 2024), frailer and had more comorbidities (Loveland et al., 2022) which can be a risk factor for increased LOS and higher readmission rates. De Stampa et al. (2023) found that older patients are more likely to be admitted to HITH, who present with significant functional impairments, with palliative care making up a substantial proportion of cases. They found that high numbers of readmissions could be explained by poor health status of patients, and having received high levels of care during previous hospitalisation was a risk factor for readmission. This was supported by another study that found older, frail patients were high users of home-based services, so it is not unexpected that these patients would have poorer outcomes such as increased readmissions (Tierney et al., 2021). A possible reason for increased LOS in home-hospital patients is that home-hospital care may be selected when longer admissions are anticipated (Montalto et al., 2020). Patients at home also experience less supervision from medical professionals. This may influence adherence to medication regimes and prescribed rehabilitation such as exercise which may have an impact on LOS and readmissions.

Overall the inconsistent results in LOS and readmission rates may be due to the type of patients the hospitals are treating and appears to be related to disease severity. Inconsistencies may also be influenced by variability in study designs, which included a mixture of RCTs, cross-sectional studies and other study designs. There are also differences in how LOS is measured, and the difference in the follow-up periods when evaluating readmission rates. Different hospitals may treat patients with various demographics. Older patients tend to be frailer and have more comorbidities (Loveland et al., 2022). Also in some studies, HITH was only available to medically stable patients (Chia et al., 2020), whereas other studies describe treating more complex patients at home, which may result higher readmission rates and longer LOS. Other inconsistencies may be due to differences in healthcare providers, such as different HITH practices, and differences in the type of patient treated by a healthcare organisation. There may also be variables not accounted for, including SES, carer availability and suitability of home environment, which may influence outcomes.

### Strengths and Limitations

This review has several strengths. The search strategy included large databases and only peer-reviewed studies were included. All articles were judged high-quality as assessed by the CASP checklist, however, the studies varied in research methodology and design, and sample size. Studies that were particularly useful for this review included randomised control trials (RCT), however, due to the nature of the study, double blinding was not possible. Several studies used a non-randomised study selection, so selection bias may be a problem (Chia et al., 2020). Many studies focused on patients with a single condition, so their results may not apply to patients with other illnesses. Also there were several studies that were 25 years old, so their results may not be generalisable to the modern healthcare system. Some studies did not control for potential confounders, such as sociodemographic factors, age groups, sex and race (Augustine et al., 2023). This review employed a narrative approach and a meta-analysis was not employed, due the differences in study design of selected articles.

## Conclusion

This systematic review highlights the benefits of home-based hospital care, and factors influencing LOS and readmission for older patients accessing this care. Home-based hospital care is an effective healthcare model, and the majority of studies found that patients participating in home-based had a shorter initial acute hospitalisation LOS than patients transferred to subacute hospital or rehabilitation ward. Overall, LOS and readmission rates (comparing home-based care to hospital care) were inconsistent but appear related to patient demographics and disease profile and require further study. By understanding factors associated with LOS and readmissions for patients at home, this may enable improvement in the delivery of home-based hospital services to older patients and help to maintain home-based hospital as a viable alternative to traditional in-hospital treatment.

## Supplemental Material

Supplemental Material - Factors Associated With Length of Stay and Readmission Rates for Older Hospital in the Home Patients: A Systematic Review Supplemental Material for Factors Associated With Length of Stay and Readmission Rates for Older Hospital in the Home Patients: A Systematic Review by Kerry de Vent, Joanne E. Porter, and Jo-ann Larkins in Journal of Aging and Health
