# Supplementary material for: Factors Associated With Length of Stay and Readmission Rates for Older Hospital in the Home Patients: A Systematic Review
Source: J Aging Health. 2025 Mar 27;38(5-6):250–60. doi: 10.1177/08982643251329425 (PMC13103338; doi:10.1177/08982643251329425)
Supplement: Supplemental Material - Factors Associated With Length of Stay and Readmission Rates for Older Hospital in the Home Patients: A Systematic Review [file sj-pdf-1-jah-10.1177_08982643251329425.pdf]

Supplementary Tables 1: Search Strategies

SCOPUS

"home hospital" OR "hospital in the home" OR hith OR "home based care" OR "hospital at home" OR HaH OR homecare
65 OR elderly OR older OR geriatric OR old* OR senior
"length of stay" OR los OR "shorter stay" OR readmi*

PUBMED

	Query	
4	#1 AND #2 AND #3	
3	"length of stay" OR los OR "shorter stay" OR readmi* OR ("Patient Readmission/standards"[Mesh] OR "Patient Readmission/statistics and numerical data"[Mesh]) OR ("Length of Stay/economics"[Mesh] OR "Length of Stay/statistics and numerical data"[Mesh] OR "Length of Stay/trends"[Mesh])	
2	65 OR elderly OR older OR geriatric OR old* OR senior OR ("Aged/physiology"[Mesh] OR "Aged/psychology"[Mesh] OR "Aged/statistics and numerical data"[Mesh])	
1	"home hospital" OR "hospital in the home" OR hith OR "home based care" OR "hospital at home" OR HaH OR homecare OR ("Home Care Services, Hospital-Based/organization and administration"[Mesh] OR "Home Care Services, Hospital-Based/standards"[Mesh] OR "Home Care Services, Hospital-Based/statistics and numerical data"[Mesh] OR "Home Care Services, Hospital-Based/trends"[Mesh])	
LIMIT TO BOOKS AND DOUCMENTS, CLINCIAL TRIAL, RCT.		

MEDLINE **CINAHL Complete APA PsycArticles APA PsycInfo**

"home hospital" OR "hospital in the home" OR hith OR "home based care" OR "hospital at home" OR HaH OR homecare
65 OR elderly OR older OR geriatric OR old* OR senior
"length of stay" OR los OR "shorter stay" OR readmi*

Filter by AGE: 65+ years   Academic journals

WEB OF SCIENCE

"home hospital" OR "hospital in the home" OR hith OR "home based care" OR "hospital at home" OR HaH OR homecare
65 OR elderly OR older OR geriatric OR old* OR senior
"length of stay" OR los OR "shorter stay" OR readmi*
